# Dental health care for children with Down syndrome: Parents’ description of their children's needs in dental health care settings

**DOI:** 10.1111/eos.12859

**Published:** 2022-02-26

**Authors:** Malin Stensson, Johanna Norderyd, Marcia Van Riper, Luc Marks, Maria Björk

**Affiliations:** ^1^ Centre of Oral Health School of Health Sciences Jönköping University Jönköping Sweden; ^2^ CHILD Research Group SIDR Jönköping University Jönköping Sweden; ^3^ National Oral Disability Centre for Rare Disorders The Institute for Postgraduate Dental Education Jönköping Sweden; ^4^ School of Nursing The University of North Carolina at Chapel Hill, North Carolina USA; ^5^ Center for Dentistry and Oral hygiene University Medical Centre University of Groningen Groningen The Netherlands; ^6^ Dept. of Special Care in Dentistry Oral Health Sciences University of Gent Gent Belgium; ^7^ Department of Nursing Science School of Health Sciences Jönköping University, Jönköping Sweden

**Keywords:** children, children's rights, dental health care, Down syndrome

## Abstract

A visit to the dental clinic may be challenging for a child with Down syndrome due to medical and oral health problems as well as communication problems. The aim of the present study was to explore how parents of children with Down syndrome describe their child's needs in the dental health care setting. In a survey concerning parental experiences with dental health care in Sweden, free comments were analysed with content analysis and resulted in five categories: “Need for continuity of care in dental health care”; “Need for dental health care professionals to have knowledge and expertise in caring for children with Down syndrome and other disabilities”; “Need for dental health care professionals to use a caring approach with children with Down syndrome”; “Need for the child with Down syndrome to be prepared to participate in their dental health care visit” and “Need for the child with Down syndrome to be given the same rights as typically developing children”. To support children with Down syndrome in an optimal way, dental health care needs to be tailored to meet the child's unique needs. In addition, dental health care professionals need knowledge of and expertise in the care of children with Down syndrome.

## INTRODUCTION

Children with Down syndrome have a higher prevalence of dental and oral conditions as well as a higher risk of medical problems with oral health consequences, which suggests that they need ongoing support from dental health care professionals [1]. In addition, visual and hearing impairments are common in children with Down syndrome, and together with intellectual disability these may impact communication in dental health care [2, 3]. Children with Down syndrome do not constitute a homogeneous group; there is a considerable variation in intellectual and physical functioning [4]. Moreover, in children with Down syndrome (as well as in children with other intellectual disabilities) there are differences in how the child is allowed to participate and be involved in their own health care. Some individuals need more support than others [4, 5]. Attitudes to and lack of knowledge about disability among health care professionals and a stereotyped image of children with Down syndrome can influence the implementation of needed health care and dental health care [6, 7].

According to the Swedish national medical guidelines for children with Down syndrome, it is recommended to have established contact with dental health care professionals from around 1 year of age at the latest. The guidelines highlight the value of cooperation between specialist and general dental health care professionals [8]. Therefore, the child's place of residence can affect the possibility of accessing and utilising dental health care. While families in some areas have well‐established access to dental health care specialists, families in northern Sweden may have limited access [9].

Many parents of children with Down syndrome emphasise the importance of a holistic approach from health care professionals, along with coordination between health care organisations [10]. There have been few studies focused on parental perceptions of dental health care for children with Down syndrome especially in Sweden. Most studies report findings from countries with different health care systems. Although studies show that parents often have a positive perception of their children's oral health [11, 12], parents and other individuals who provide support to young people with disabilities have also reported that nobody takes the overriding responsibility for the young person's dental health care [13]. In addition, they have pointed out that dental health care professionals need to have knowledge and expertise in providing care to children with intellectual disabilities [14]. There is considerable variation in competence and engagement of professionals in providing dental health care for children with disabilities. Dental health care professionals and dental students have reported that they are not always comfortable in treating individuals with intellectual disabilities, because they lack knowledge and experience [15, 16, 17]. Children with Down syndrome deserve the same quality of health care as other children [18], and the same is true for dental health care. Thus, it is important to further explore Swedish parents’ perception of their children's needs when visiting the dental setting. Therefore, the aim of this study was to describe how parents of children with Down syndrome perceive their child's needs within the dental health care setting.

## MATERIAL AND METHODS

This study is based on the qualitative findings from a study of parental perceptions of oral health, general health and dental health care for children with Down syndrome in Sweden. Stensson and co‐workers presented the quantitative findings in 2020 [19]. Given that the material and methods for the main study already have been described in detail by Stensson and co‐workers [19], only a brief overview will be provided here.

Parents of children with Down syndrome between 0 and 18 years of age were invited to the study through the Swedish Downs Syndrome Society's Facebook page and their website (www.svenskadownforeningen.se) in the first 3 months of 2018. At that time, the Swedish Down Syndrome Society had approximately 480 members. The study involved completing a 50‐item survey with both closed and open‐ended questions about parental perceptions of general health, oral health and received dental health care in children with Down syndrome.

In the 50‐item survey [19], the respondents could make free comments. These comments were related to the dental health care, skills and knowledge of dental health care professionals and the encounter between the child and the professionals.

### Data analysis

Parental responses to the open‐ended questions in the questionnaire (92 free‐text answers) were analysed with content analysis utilising the three main phases outlined by Elo and Kyngäs [20]. In the first phase, the preparation phase, two of the authors (MB, MS) read the free‐text answers several times to obtain a sense of the content. When a sense of the content had been established, meaning units, that is, text related to the aim of the study—were highlighted, extracted from the questionnaire and pasted into a separate document. In the second phase, the organisation phase, meaning units were sorted under different preliminary generic categories using an inductive approach. Subcategories were then identified based on similarities and differences of content. To reduce the number of categories, data were grouped together by collapsing subcategories that could be gathered under the same content. There was a continuous movement back and forth between categories and an ongoing discussion between the authors until a consensus was reached. In the third phase, the reporting phase, the results from the analysis were grouped into five categories.

### Ethical considerations

Participants were informed that completion of the questionnaire was voluntary, and that data would be confidential. The parents consented to their participation in the research study by answering the questions in the questionnaire. In accordance with the World Medical Association Declaration of Helsinki, ethical approval was obtained from the Regional Ethics Committee for Human Research at Linköping University, Sweden 2016/425‐31.

## RESULTS

In total, 101 parents participated in the study (91 mothers and 10 fathers). They were aged between 28 and 58 years (mean age 42.8), and approximately 70% had a university/college degree. Their children's age ranged from 1 to 19 years (mean age: 9.6 years); 53 (52%) were boys and 49 (48%) were girls. Most of the children lived with both of their parents in the more populated parts of Sweden, the middle and the south. Approximately 50 (∼50%) of the children attended elementary special schools; 35 (∼35%) attended elementary schools, and 16 (∼16%) were below school age. The majority of the parents used the Swedish language (94%), and 20% used signs and/or pictures to support communication with their child with Down syndrome.

Five categories of need were identified:

- Need for continuity of care in dental health care;
- Need for dental health care professionals to have knowledge and expertise in caring for children with Down syndrome and other disabilities;
- Need for dental health care professionals to use a caring approach with children with Down syndrome;
- Need for the child with Down syndrome to be prepared to participate in their dental health care visit; and
- Need for the child with Down syndrome to be given the same rights as typically developing children.

Each category is described below:

### Need for continuity of care in dental health care

Many parents stated that there was a lack of continuity of care in the dental health care provided to their child with Down syndrome. One parent stated: *“Lack of continuity. My child does not have a dedicated dentist and meets new dentists every time when being at the dental health care clinic”*. Parents noted that even if their child with Down syndrome did not usually have problems meeting new people, they may need extra time to adjust to a new dental health care professional, since meeting new staff might remind them of previous dental health care visits that were stressful or frustrating. Visits to dental health care professionals tended to go better if there was continuity from one visit to the next. One parent wrote: “*He always visits the same dental hygienist and dentist, which is superb. I also appreciate that he is allowed to visit them several times a year. However, during the years, it has not always been good”*.

### Need for dental health care professionals to have knowledge and expertise in caring for children with Down syndrome and other disabilities

Parents indicated that many dental health care professionals seemed to lack knowledge about, and experience in, providing dental health care to children with intellectual disabilities in general, but also dental health issues specific to children with Down syndrome. Because of this, their child with Down syndrome did not always receive the care they needed. Additional visits were sometimes necessary, or in some circumstances the child had to switch to another dental health care professional. Having to switch to another dental health care professional often lead to a prolonged period of time before the child's next appointment. One parent wrote: *“Our dentist terminates the examination if my son is showing a troublesome behaviour. Then she will not even try. We will change dentists as we have visited her twice and at both times, we had to go home without having the teeth examined as the dentist has not even tried. She assumes that my son won't make it!”*. Some parents noted that it seemed as if, prior to meeting with the child with Down syndrome, the dental health professional had already decided that the child would be unable to handle different kinds of examination.

Many parents reported that the professionals lacked knowledge of the unique communication needs of children with intellectual disabilities, as well as how to use alternative ways to enhance communication, such as signs and pictures as a support. Parents thought that the visit would have been more successful if the dental health care professionals had used pictures and signs. One parent wrote: “*It would have been so helpful if they (the dental health care professionals) used and communicated with help of pictures and signs in combination with spoken language”*.

Parents reported that when dental health care professionals had difficulty understanding both the verbal and body language of the child with Down syndrome, as well as the child's needs, it was important for someone who knew the child to accompany them in order to interpret the child's thoughts and feelings, as well as to help the child to feel safe. One parent wrote: *“Most of them don't understand that they have to wait for my son, they have to give him time to think, time to understand and time to process what he has heard to give him time to prepare an answer. Many of them also think that they understand what he means, but in reality, they have not understood a thing. This implies that he always has to have someone with him that knows him, how he reacts and what he means”*.

### Need for dental health care professionals to use a caring approach with children with Down syndrome

Parents stressed that it was important for dental health care professionals to meet the child with Down syndrome in an open and sensitive way. Parents offered many specific suggestions for how dental health care professionals should approach children with Down syndrome and remarked that it was important for professionals to be calm, patient, humble and without prestige, otherwise it could lead to difficulties. One parent wrote: *“Before, we went to a specialist dentist and it did not go well because they were not willing to compromise and were not flexible about how the visit should go—the professionals decided how things would go. My daughter immediately felt that she was not allowed to decide anything and therefore she refused to sit in the chair. It became a fight about EVERYTHING. However, the dentist we have now is open and sensitive towards my daughter and now she is not at all recalcitrant. It is like night and day! Very interesting!”*. Another parent wrote, *“If she just feels a little bit that the professional want to “govern” her, it makes her refuse. She wants to feel that she is in charge and that she has control over the situation”*.

Some parents stressed that it was important for dental health care professionals to talk directly to the child and to be clear and open with what they wanted the child to do and what the child could expect. Parents indicated that there was no use in forcing the child, instead the dental health care professional needed to explain things to the child with Down syndrome so that the child could agree to participate. One parent, whose child had positive interactions with dental health care professionals, wrote: *“They give him a lot of time. He can touch everything they have (and they have as you know a great deal of stuff) before they ask him to sit down. It did not work as good when his dentist was stressed last time so I hope it will be better next time”*.

### Need for the child with Down syndrome to be prepared to participate in their dental health care visit

Some parents indicated that their children with Down syndrome were very determined in their view of how things should be, which could be aggravating when visiting the dental health care professional. One parent wrote that *“He usually is very determined that they are not allowed to give him an injection or look in his mouth. However, he does not get upset, but just says that they are not allowed to do it and he thinks that it should be that way”*. Some parents suggested it may be good to let children with Down syndrome have practice visits where the child is allowed to sit in the dental chair, is asked to open their mouth and encouraged to allow the dental health care professional to examine their teeth. One parent related: *“…and to practice going to the dentist as he rather doesn't want to open his mouth. Last time they could actually count the teeth in the lower jaw!”*.

Some of the parents reported that they had asked for preparational/practice visits, but this was not offered. Other parents noted that they wanted to book a visit at the dental clinic without the child before the child's scheduled visit. They wanted to come alone to discuss possible strategies with the dental health care professionals, as well as to speak about the time of day that possibly worked best for the child. It was a way of preparing both themselves and the dental health care professionals and gave the parent an opportunity to prepare the child before the visit.

Even when parents tried to prepare the child for a visit to the dentist, it still could be difficult for the dental health care professional to perform the examination or treatment. One parent said: *“As my son is afraid of the examination lamp in the ceiling, it is hard for him to sit in the dental chair. The dentist then must crawl down to him on the floor and do the examination there. As you understand, it is impossible to examine the teeth in a good way and, if necessary, to pull out milk teeth that don't fall out by themselves”*. Therefore, when these difficulties could not be solved otherwise, some children had to receive dental treatment under general anaesthesia.

### Need for the child with Down syndrome to be given the same rights as typically developing children

Some parents reported that they had interacted with dental health care professionals who had difficulty viewing the child with Down syndrome as a unique individual. Instead, the dental health care professional seemed to assume that all children with Down Syndrome were alike. Because of this, the dental health care professional behaved differently with their child with Down syndrome than they did when meeting typically developing children. One parent wrote: *“The problem always lies in the hand of the other. My son has no problem meeting people, but those that he meets often have a problem with meeting him”*.

Some parents questioned if their child with Down syndrome was treated in the same way as a child with typical development would have been and they stressed the rights of every child. Parents noted that some dentists did not seriously consider the fact that tooth agenesis is more common in children with Down syndrome, and this needs to be taken into consideration. Some also experienced situations that gave them the view that the dentist did not think that aesthetics–having nice‐looking teeth–is as important for children with Down syndrome as it is for typically developed children.

“I have an uncomfortable suspicion that they have a bias like “she had Down Syndrome and then it is not that important how she looks like in the mouth”, but I do not know this for sure. I have capabilities and financial resources and will make sure that my daughter receives correct cosmetic treatment in the future, exactly like a typically developed teenager would have demanded, but all parents don't have that ability. Dental health care for children should be equal, especially important for children that will have limited financial resources to do extended cosmetic dental treatments as adults”.

## DISCUSSION

This paper reports the free‐text answers from a survey about how Swedish parents of children with Down syndrome perceive their child's needs within the dental health care setting. Five categories of need were identified. The first was the need for continuity of care in dental health care. The next two needs were focused on health care professionals and dental health care professionals in particular; they need to have knowledge and expertise in caring for children with Down syndrome and other disabilities and they need to use a caring approach. The last two needs were focused on the child with Down syndrome. Children with Down syndrome need to be prepared to participate in their dental health visit and they need to be given the same rights as typically developing children.

Our findings indicate that parents of children with Down syndrome express a lack of continuity in the dental health care that their child with Down syndrome receives. Consequently, the child needs extra time to adjust to new dental health care professionals. Continuity of care is important for all children, but it is especially important for children with Down syndrome and other chronic conditions. Continuity of care can be enhanced if there is care coordination. The latter is the management of care provided by members of the healthcare team from a variety of settings [21]. Children with Down syndrome are at greater risk of missed care coordination than other children with chronic conditions [22, 23].

In a recent scoping review of care coordination needs of families of children with Down syndrome, Skelton and colleagues [24] identified three needs: communication, information and utilisation of health resources. Additional needs focused on individual, family and healthcare contextual factors. Individual and family factors included experience with healthcare, individual functioning and family functioning. Healthcare contextual factors included resources, technology use and dental health care. Five of the 38 studies reviewed focused exclusively on dental care [12, 25–28].

To facilitate involvement of children with Down syndrome in their own dental health care and to make the environment more adaptive, professionals need experience and certain skills. Parents in the present study suggested that dental health care professionals need additional expertise in caring for someone with Down Syndrome, as well as knowledge in communicating with someone who has communication difficulties. In the literature, there is growing evidence that, when indicated, it is beneficial to use alternative augmentative methods of communication during examination or treatment procedures [29, 30].

One way to facilitate the ability of dental and other health care professionals’ communication with children with Down syndrome is to develop a partnership with the child and his/her parents or caregivers [31]. They are the people most likely to know the child's preferred mode of communication. Moreover, they are also likely to know what kind of strategies will work best to get their child to cooperate in different situations. Health care professionals who work in partnership with the child and their parents or caregivers are more likely to feel prepared to care for and to communicate with the child.

There seems growing evidence that many dental and other health care professionals feel unprepared to care for and communicate with individuals with Down syndrome and other disabilities, and, because of this, some may refer the child to a specialist [4, 17, 22, 32]. This is not surprising, given the findings from a study of how health care professionals are prepared to provide care to individuals with disabilities [32]. In that study, more than half (58%) of the responding deans of medical schools and 50% of the deans of dental schools reported that a curriculum for patients with disabilities was not a high priority. While 58% of the former and 47% of the latter reported their graduates were competent in treating patients with disabilities, most of the medical and dental school seniors and graduates expressed their inadequacy in caring for individuals with disabilities.

In a review of studies focused on acute care nurses’ experiences with patients who had an intellectual disability [33], three themes were identified: (1) nurses feel underprepared when caring for individuals with intellectual disabilities; (2) they experience challenges when communicating with individuals with intellectual disabilities, and (3) they have ambiguous expectations of paid and unpaid caregivers. These findings provide support for our observation that dental and other health care professionals need better preparation in how to care for and communicate with people with intellectual disabilities. Lewis and colleagues [33] recommended that such preparation should be twofold and include considering the complexities of communicating with individuals with intellectual disabilities. Obtaining practical experience of doing so in clinical and educational environments ensure the safety and dignity of people with intellectual disabilities and the health care professionals who care for them. They argue that, although communication with people with intellectual disabilities can be very time‐consuming, merely increasing the amount of time allotted is not enough [33]. Instead, greater attention needs to be paid to assessing the preferred mode of communication with each such individual. For example, instead of just trying to communicate verbally, it may be more effective to use alternative methods of communication because some individuals may respond better to nonverbal modes of communication or verbal communication augmented by symbols and pictures.

Our findings suggest that it is important for dental health care professionals to meet the child with Down syndrome in an open and a sensitive way. Dental examination and dental treatment can be a challenge both for the child as the professionals, and professionals’ adaptation to the child's level of understanding is important. The success of this adaption highly depends on the dental health care professional's ability to meet and involve the child. Parents of children with Down syndrome valued dental health care professionals who were patient and had an ability to engage the child [19], as well as those who were sympathetic, good communicators and well‐informed about Down syndrome [6]. Parents are more satisfied when they can effectively collaborate with the professionals, which, they argued, improved their child's quality of life [34]. The attitude of professionals can affect how care is provided, and a stereotyped image of children with Down syndrome may negatively influence the care they receive. A negative experience can increase the risk of frustration in the child and the parents and may lead to uncooperating behaviour in the child [35].

Parents of children with Down syndrome have expressed a need for appropriate oral health information early in their child's life [36]. This was also true in the present study. In addition, parents in this study wanted tools or strategies to help prepare their child to participate in their dental health care visit. For example, some parents wanted contact with the dental health care professionals before the dental visit to receive information about the visit as well as to provide information about their child. In the process of preparing and involving a child in their own care, parents’ support is important and will affect the child's ability to cope with the situation [37]. Information provided by a parent to a child prior the child's visit can improve the child's participation. Moreover, if children receive adapted information and are allowed to participate, they may also make competent decisions themselves [30, 38, 39, 40]. Utilising dental health care, both general and specialised, can be described in terms of participation. In the present study, parents expressed the need for their children's participation in their own dental health care. According to the fPRC framework created by Imms and colleagues in 2017 [5], participation has two dimensions: attendance/being there, and involvement while being there. Children who experience a high degree of participation report stronger autonomy and higher wellbeing than those who do not experience the same level of participation. In the fPRC framework, participation is dependent on the child's and care providers’ characteristics, the environment's adaptability and how well a positive context is co‐constructed by the child and the environment.

Children with Down syndrome may require adaptations in the care process, such as communication support, in order to be fully participative. The results from our study question whether this is actually applied. Even if children with Down syndrome may face barriers to receiving dental treatment, they should be given support in order to provide them with the same possibilities as children without disabilities.

One limitation of the present study is that the free‐text answers are not representative of all parents in the study. Those who have added free‐text answers are likely to be the most motivated parents. Notwithstanding, the study contributes to the general research landscape by providing some parents’ perspectives. However, future research should investigate the children's own views and specific needs during dental visits. To conclude, many children with Down syndrome need to visit dental health care services regularly. When visiting dental health care services, they need continuity, and they need to meet professionals who have a caring approach and knowledge and experience of children with Down syndrome. The children also need to participate in their own dental health care visit, which calls for preparation. These results may also be transferred to other groups of children that need special support. However, a child's medical condition and intellectual disability may constitute a barrier to accessing required and necessary treatment. In order to provide the best dental health care, it is therefore important to focus on each child's individual needs and to see them as active participants in their own care.

## CONFLICTS OF INTEREST

The authors have no conflicts of interest relevant to this article.

## AUTHOR CONTRIBUTIONS

**Conceptualization**: Malin Stensson, Luc Marks, Maria Björk; **Methodology**: Maria Björk, Malin Stensson; **Software**: Maria Björk, Malin Stensson; **Formal analysis**: Maria Björk, Malin Stensson, Johanna Norderyd, Marcia Van Riper, Luc Marks; **Investigation**: Maria Björk, Malin Stensson, Johanna Norderyd, Marcia Van Riper, Luc Marks; **Writing ‐ original draft preparation**: Maria Björk, Malin Stensson, Johanna Norderyd, Marcia Van Riper, Luc Marks; **Project administration**: Malin Stensson; **Funding acquisition**: Malin Stensson.
